# Effect of inactivated COVID-19 vaccination on intrauterine insemination cycle success: A retrospective cohort study

**DOI:** 10.3389/fpubh.2022.966826

**Published:** 2022-09-12

**Authors:** Zijin Xu, Yixuan Wu, Yanshan Lin, Mingzhu Cao, Zhu Liang, Lei Li, Jiali Lin, Qian Chen, Jianqiao Liu, Haiying Liu

**Affiliations:** ^1^Department of Obstetrics and Gynecology, Center for Reproductive Medicine, Guangdong Provincial Key Laboratory of Major Obstetric Diseases, The Third Affiliated Hospital of Guangzhou Medical University, Guangzhou, China; ^2^Key Laboratory for Reproductive Medicine of Guangdong Province, The Third Affiliated Hospital of Guangzhou Medical University, Guangzhou, China

**Keywords:** COVID-19, inactivated vaccine, SARS-CoV-2, IUI, infertility

## Abstract

**Background:**

Vaccine hesitancy was found in couples seeking artificial reproductive technology (ART) services. As the main vaccine used in China, investigations into the influence of inactivated coronavirus disease 2019 (COVID-19) vaccines on human fertility is needed.

**Methods:**

This retrospective cohort study included data on COVID-19 vaccination, clinical characteristics, and reproductive outcome of 1,000 intrauterine insemination (IUI) cycles in 653 couples from March 2021 to March 2022 in a single university hospital-based center for reproductive medicine. The IUI cycles were divided into two categories based on sperm source, including 725 cycles in 492 women undergoing artificial insemination with their husband's sperm (AIH) and 275 cycles in 161 women undergoing artificial insemination with donor sperm (AID). Women were then divided into two groups. The vaccine exposed group included women vaccinated prior to insemination and the unexposed group included women who were not vaccinated or vaccinated after insemination. Reproductive outcomes including ongoing pregnancy rate, clinical pregnancy rate, and miscarriage rate were assessed.

**Results:**

Inactivated COVID-19 vaccinated women prior to intrauterine insemination in AIH cycles have comparable ongoing pregnancy rate (11.1 vs. 10.3%, *P* = 0.73), clinical pregnancy rate (12.5 vs. 11.3%, *P* = 0.60) as compared with unvaccinated counterparts. Similarly, there were no significant differences in ongoing pregnancy rate (20.9 vs. 28.1%, *P* = 0.17), clinical pregnancy rate (21.7 vs. 28.8%, *P* = 0.19) between vaccine exposed and unexposed groups in AID cycles. Multivariable logistic regression analyses showed that inactivated COVID-19 vaccination status cannot independently influence the reproductive outcomes of AIH and AID cycles. Subgroup analysis of vaccine exposed cycles showed that doses of vaccination and Interval between the last dose of vaccination and insemination have no influence on the reproductive outcomes of AIH cycles.

**Conclusions:**

No negative effects were found on female fertility in IUI cycles following exposure to the inactivated COVID-19 vaccine. These findings indirectly reflect the safety of inactivated COVID-19 vaccine toward reproductive health and help to mitigate vaccine hesitancy among people planning to conceive.

## Introduction

The outbreak of Coronavirus disease 2019 (COVID-19) has developed into a global pandemic recognized by the World Health Organization (WHO) on the 11th of March 2020 and continues to pose a great threat to public health and safety (1). As of March 2022, over 455 million confirmed cases and almost 6 million deaths had been reported globally (2). COVID-19 is caused by severe acute respiratory syndrome coronavirus 2 (SARS-CoV-2) wild-type strain and its variants, a novel positive-stranded RNA virus belonging to the Coronaviridae family (3). Because of the vulnerability of SARS-CoV-2, the development of safe and effective vaccines has become the most urgent goal for the scientific community. Globally, various vaccines are being developed, including live-attenuated virus vaccines, inactivated virus vaccines, protein subunit vaccines, replication-deficient vectors, and genetic vaccines (DNA and RNA) (4). According to data from the WHO, there are at least 149 vaccine candidates in clinical phases, 40 of which have reached Phase III trials based on different vaccine platforms (5). Inactivated vaccines are the most commonly used in China because three double-dose inactivated vaccines (Sinovac and SinoPharm) have been approved for emergency use. After being adopted in a nationwide anti-COVID-19 vaccination program, over 3,100 million doses of inactivated vaccines were administered in mainland China (6).

Studies have shown that the SARS-CoV-2 virus initiates infection through the interaction of its spike proteins with the human angiotensin-converting enzyme 2(ACE2) receptors (7), which are abundant in the ovarian and testicular tissue of the human reproductive system (8, 9). This highlights the potential for detrimental effects on the future fertility of people infected with SARS-CoV-2. It is also particularly concerning for the uptake of COVID-19 vaccines, given its importance for people of child-bearing age. Based on our understanding of the immune response to inactivated vaccines and the efficacy and safety data from clinical trials (10–13), current guidelines from various world organizations do not restrict COVID-19 vaccination from people trying to conceive or undergoing ART. However, given the lack of information on the specific effects of COVID-19 vaccination on reproduction, there is no consensus on the need to postpone conception after vaccination. Guidelines from the European Society of Human Reproduction and Embryology (ESHRE) and the Chinese Expert Group recommend postponing ART for at least a few days after administration of the vaccine to allow the immune response to settle (14). Conversely, other organizations such as the American Society for Reproductive Medicine (ASRM), the Centers for Disease Control and Prevention (CDC), and the Joint Committee on Vaccination and Immunization (JCVI) do not stress this point (15). Additionally, we found that couples seeking artificial reproductive technology (ART) services in our reproductive center focused more on the effect of COVID-19 vaccination on ART and future pregnancy, which led to vaccine hesitancy and extremely low vaccination coverage.

Intrauterine insemination (IUI) also known as artificial insemination, is the first-line treatment for unexplained and male-factor infertility. With this treatment, the sperm from a partner or donor is prepared and inseminated directly into the uterus around the time of ovulation, representing the relatively natural fertilization process compared to *In vitro* fertilization embryo transfer (IVF-ET) (16). Therefore, this study aims to identify the effect of inactivated COVID-19 vaccines on reproductive outcomes in a cohort of women undergoing IUI cycles to increase confidence and reduce hesitancy toward these vaccines in women trying to fall pregnant.

## Materials and methods

### Study design and participants

**Figure 1:**
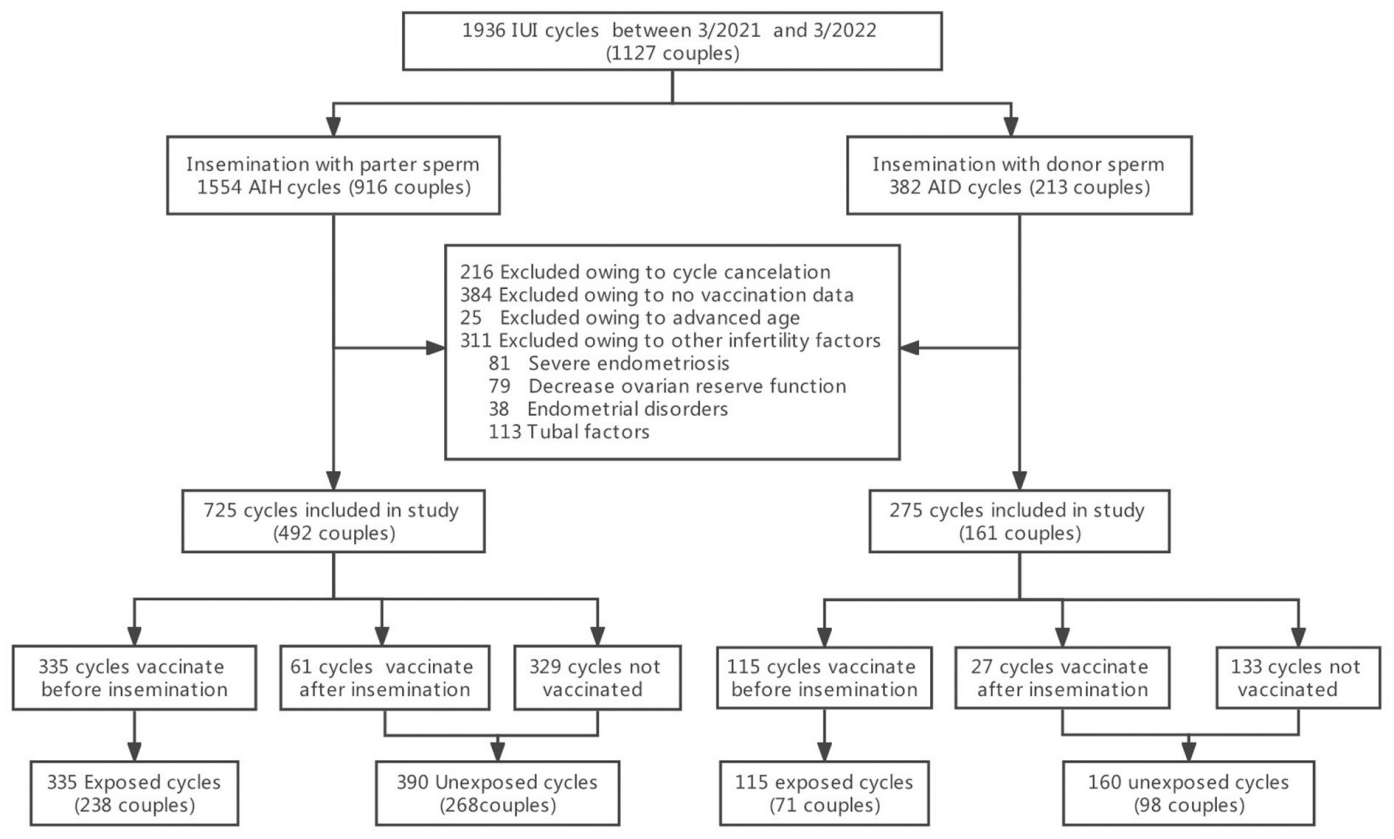
Study flowchart.

A retrospective cohort study was conducted at the Center for Reproductive Medicine of the Third Affiliated Hospital of Guangzhou Medical University (Guangzhou, China). Women who had undergone IUI cycles from March 2021 to March 2022 were enrolled. Inclusion criteria included: (i) at least 12 months of infertility, (ii) regular menstruation (21–35 days), and (iii) normal uterine cavity with at least one patent fallopian tube (established by hysterosalpingography or laparoscopy). Exclusion criteria included: (i) advanced maternal age (older than 40 years), (ii) no COVID-19 vaccination data, (iii) cycle cancellation due to a low ovarian response (lack of development of lead follicle at least >14 mm), ovulation from the side of known tubal occlusion, multifollicular response and premature ovulation, and (iv) presence of other infertility factors including severe endometriosis e(ASRM grade III-IV), decreased ovarian reserve function (antral follicular count (AFC) <5–7 follicles or anti-Müllerian hormone (AMH) <0.5–1.1 ng/ml), endometrial disorders (polyps or submucosal fibroids) and hydrosalpinx. From the 1,127 infertile couples identified (1,936 cycles), 916 women (1,554 cycles) underwent artificial insemination with their husband's sperm (AIH), and 213 women (382 cycles) underwent artificial insemination with donor sperm (AID), primarily due to severe male factor infertility (17). These people were further screened by the above exclusion criteria. Finally, 725 AIH cycles and 275 AID cycles were included in the study and each was divided into two groups. The vaccine exposed group included women vaccinated prior to insemination and consisted of 335 AIH cycles and 115 AID cycles. The unexposed group included women who were not vaccinated or vaccinated after insemination and consisted of 390 AIH cycles and 160 AID cycles (Figure 1).

The baseline clinical characteristics and cycle variables were collected from a fertility department database. Vaccination status was determined by telephone follow-up. General patient information such as female age, body mass index (BMI), type of pregnancy, infertility duration, treatment cycle type, IUI indication, and cycle number was recorded. The indications for IUI were divided into male factors, unexplained or other factors, while treatment cycle types were divided into cycles with controlled ovarian stimulation (COS) and natural cycles. Cycle variables cover an index that reflects ovarian function, including basal follicle-stimulating hormone (FSH) level, AMH level, and bilateral AFC; male sperm parameters including progressive motility (PR) after processing and the total motile sperm count (TMSC) after processing; and the numbers of dominant follicles and endometrial thickness on the day of hCG administration. Vaccination status included the male partner vaccinated or not, the doses of vaccination, and the interval between the last dose vaccination and insemination in exposed cycles.

This study was approved by the local Ethics Committee of the Third Affiliated Hospital of Guangzhou Medical University.

### IUI protocol

Details of the IUI protocol have been described previously (18). A transvaginal ultrasound was performed on cycle day 35 to exclude ovarian cysts larger than 30 mm. According to the maternal age and ovarian reserve testing, the women started intramuscular injections of human menopausal gonadotropin (HMG, Livzon, Zhuhai, China), ranging from 37.5 to 75 IU to control ovarian stimulation. These injections continued daily until ovulation of at least one follicle ≥17 mm in diameter.

The trigger criteria for ovulation were: (i) the leading follicle was ≥17 mm in diameter, (ii) the serum luteinizing hormone (LH) was elevated and the leading follicle was at least 14 mm in diameter, (iii) the serum P concentrations were ≥1.5 pg/l and the leading follicle was at least 14 mm in diameter. If one of these three criteria were observed, ovulation was triggered with human chorionic gonadotropin (hCG) ranging from 5,000 IU to 10,000 IU.

Insemination was performed 12–36 h after hCG injection. The sperm was collected by masturbation after 2–7 days of sexual abstinence. Sperm samples in the AID cycles were obtained from the Human Sperm bank of Guangdong Province. Sperm from each washed semen sample were counted and evaluated for motility, and 0.2–0.5 mL was introduced into the woman's uterus by syringe.

Ovulation was identified by free fluid in the Douglas pouch and visible corpus luteum and/or the disappearance of the follicle during a transvaginal ultrasound. After insemination, micronized progesterone (200 mg vaginal capsule, twice daily) was used for luteal support. The serum β-HCG level was tested for pregnancy 2 weeks later.

The primary response variable for this study was the ongoing pregnancy confirmed by intrauterine pregnancy beyond 12 weeks' gestation through transvaginal ultrasound examination, and clinical pregnancy defined as the presence of a yolk sac with heartbeat at 7 weeks gestation. Secondary outcomes included rates of biochemical pregnancy, early miscarriage, and ectopic pregnancy. Biochemical pregnancy was determined as the detection of serum level of HCG more than 10 mIU/ml 14 days after operation. Biochemical pregnancy loss was determined as elevated HCG levels but no detectable gestational sac was observed with transvaginal sonography 4 weeks following operation. Early miscarriages were those pregnancy losses with detectable intrauterine gestational sacs within gestational 12 weeks. Ectopic pregnancy was identified as embryos implant at any other sites except for intrauterine cavity. Biochemical pregnancy loss rate, spontaneous miscarriage rate, and ectopic pregnancy rate were calculated based on the number of women with biochemical pregnancy.

### Statistical analysis

The mean ± standard deviation (SD), median and interquartile range (IQR 25 to 75%) were determined for continuous variables, while categorical variables were expressed as cycle numbers and percentages. A Mann-Whitney U-test was used to compare the response variables between groups for skewed data, and a *t*-test was used for normally-distributed data. A chi-square test was used to compare qualitative data between groups. Clinical pregnancy, biochemical pregnancy, and miscarriage rates were compared for vaccine-exposed or unexposed groups in AIH and AID cycles. First, the unadjusted risk ratio (RR) and 95% confidence interval (CI) were calculated for clinical pregnancies, using unexposed cycles as the reference. A log-binomial regression model for multivariate analysis was then performed, controlling for female age, BMI, infertility duration, treatment cycle type, IUI indication, sperm parameters after processing, ovarian reserve function, dominant follicles, endometrial thickness on the day of hCG administration, and the vaccination status of male partner. Next, a generalized estimating equation (GEE) was used to examine the relationship between individual factors and the outcome of ongoing pregnancy, controlling for multiple cycles within the same couple. RR and 95% CI were calculated for candidate factors. A *p*-value of < 0.05 indicated statistical significance. Statistical analysis was performed in SPSS 28.0 software (IBM).

## Results

From March 2021 to March 2022, data from 1,000 IUI cycles in 653 couples were included in this study, of which 725 were cycles with partner sperm (492 couples) and 275 were cycles with donor sperm (161 couples). There were 335 AIH cycles in the COVID-19 vaccine-exposed group and 390 cycles in the unexposed group. Similarly, 115 AID cycles were in the COVID-19 vaccine-exposed group, and 160 cycles were in the unexposed group. Table 1 summarizes baseline characteristics per artificial insemination cycle stratified by vaccine exposed or not. The mean female age was 31.2 ± 3.6 years in AIH cycles and 29.6 ± 3.5 years in AID cycles. There were no statistically significant differences in the female age, BMI, infertility duration, distribution of infertility types, treatment cycle types, IUI indication, or cycle number between vaccine-exposed and unexposed groups in AIH cycles (*P* > 0.05). In AID cycles, women with vaccine exposure were significantly older than those unexposed (30.5 vs. 29.4, *P* = 0.02).Other baseline characteristics did not differ significantly. The vaccination coverage of women seeking for IUI treatments was 45%. The vaccine coverage rate of male partner in the female vaccine-exposed group was significantly higher than in the unexposed group in AIH cycles (98.2 vs. 51.6%, *P* < 0.01).

**Table 1:** Baseline characteristics per artificial insemination cycles with husband or donor semen stratified by vaccination exposed or not.

**Variables**	**AIH cycles**			**AID cycles**		
	**Exposed group**	**Unexposed group**	***P-*value**	**Exposed group**	**Unexposed group**	***P-*value**
No. of cycles	335	390		115	160	
Female age, mean (SD), y	31.2 (3.8)	31.2 (3.6)	0.95	30.5 (2.4)	29.4 (3.8)	0.02
BMI, mean (SD), kg/m^2^	21.9 (3.3)	22.0 (3.4)	0.74	22.0 (2.7)	21.2 (2.8)	0.09
Type of infertility, *n* (%)			0.87			0.09
Primary infertility	218 (65.9)	252 (65.3)		102 (88.7)	130 (81.3)	
Secondary infertility	113 (34.1)	134 (34.7)		13 (11.3)	30 (18.8)	
Infertility duration, median, median (IQR), y	3 (2–4)	3 (2–4)	0.53	4 (2–5)	4 (2–5)	0.67
Treatment cycle type, *n* (%)			0.55			0.08
Natural	169 (50.4)	188 (48.2)		73 (66.1)	89 (55.6)	
COS	166 (49.6)	202 (51.8)		39 (33.9)	71 (44.4)	
IUI indication, *n* (%)			0.06			0.63
Unexplained/other	146 (44.6)	199 (51.6)		4(3.5)	4 (2.5)	
Male factors	185 (55.4)	187 (48.4)		111 (96.5)	156 (97.5)	
Cycle number, *n* (%)			0.06			0.08
First	197 (58.8)	253 (64.9)		56 (48.7)	60 (37.5)	
Second	117 (34.9)	127 (32.6)		40 (34.8)	49 (30.6)	
Third or more	21 (6.3)	10 (2.6)		19 (16.6)	51 (31.8)	
Male partner vaccination, *n* (%)	326 (98.2)	197 (51.6)	<0.01			

Table 2 shows the vaccination status of vaccines exposed group both in AIH and AID cycles. There were no statistically significant differences between AIH and AID cycles in the distribution portion of the vaccination doses as well as the interval between the last dose vaccination and insemination (*P* > 0.05). For women vaccinated before insemination, Over 70% of them have been vaccinated double doses before undergoing intrauterine insemination, and the interval between the last dose vaccination and insemination was more than 3 months.

**Table 2:** Vaccination status of vaccines exposed group.

**Variables**	**AIH cycles**	**AID cycles**	***P-*value**
Doses of vaccination, % (*n*)			0.33
Single dose prior to insemination	23.3 (78/335)	27.8 (32/115)	
Double doses prior to insemination	72.5 (243/335)	70.4 (81/115)	
Three doses prior to insemination	4.2 (14/335)	1.8 (2/115)	
Interval between the last dose and insemination, %(*n*)			0.73
<3 months	27.8 (93/335)	26.1 (30/115)	
≥3 months	72.2 (242/335)	73.9 (85/115)	

There were no statistically significant differences between vaccine-exposed and unexposed groups in the indexes representing female ovarian function, sperm parameters after processing, dominant follicles, or endometrial thickness on the day of hCG administration in AIH cycles (*P* > 0.05). The only significant difference found in AID cycles was PR after processing, which was higher in the exposed group than in the unexposed group (84.2 vs. 78.1%, *P* < 0.01, Table 3).

**Table 3:** Cycle variables per artificial insemination cycles with husband or donor semen stratified by vaccination exposed or not.

**Variables**	**AIH cycles**			**AID cycles**		
	**Exposed group**	**Unexposed group**	***P-*value**	**Exposed group**	**Unexposed group**	***P-*value**
Ovarian reserve function, median (IQR)						
Basal FSH level, mIU/mL	5.65 (5.0–6.90)	5.68 (4.87–6.46)	0.61	5.03 (4.75–6.71)	5.30 (4.54–6.27)	0.71
AMH level, ng/mL	4.15 (2.79–5.88)	4.21 (2.75–6.56)	0.17	3.66 (2.61–5.53)	3.99 (2.48–6.41)	0.84
Bilateral AFC	19 (15–25)	20 (15–26)	0.83	19 (14–29)	20 (16–25)	0.25
Sperm parameters, median (IQR)						
PR after processing, %	93.8 (91.3–96.2)	94.0 (90.5–96.0)	0.38	84.2 (75.6–87.7)	78.1 (72.6–82.8)	<0.01
TMSC after processing, 10^6^	26.44 (17.19–39.97)	29.39 (16.74–44.63)	0.51	13.39 (11.07–15.99)	12.86 (11.15–15.47)	0.13
Dominant follicles, median (IQR)	1 (1)	1 (1)	0.98	1 (1)	1 (1, 2)	0.16
Endometrial thickness on the day of hCG administration, median (IQR), mm	9.4 (8.6–11)	9.8 (8.4–11)	0.93	9.0 (7.3–10.0)	9.5 (8.8–10.4)	0.11

Table 4 shows the frequencies and adjusted RR for reproductive outcomes of artificial insemination cycles stratified by vaccine-exposed or unexposed. In AIH cycles, there were no significant differences in reproductive outcomes between groups (11.0 vs. 10.3% for ongoing pregnancy rate, *P* = 0.73; 12.5 vs. 11.3% for clinical pregnancy rate, *P* = 0.60). The rates of biochemical pregnancy (13.1 vs. 12.8%, *P* = 0.90) and biochemical pregnancy loss (4.5 vs. 12.0%, *P* = 0.28) were similar in the vaccine exposed group compared with the unexposed group. In AID cycles, the rates of reproductive outcomes were slightly lower in the exposed group, but this difference was not statistically significant (20.9 vs. 28.1% for ongoing pregnancy rate, *P* = 0.17; 21.7 vs. 28.8% for clinical pregnancy rate, *P* = 0.19; 22.6 vs. 30.6% for biochemical pregnancy rate, *P* = 0.14). Multivariable logistic regression analyses showed no independent influence of vaccine exposed on the reproductive outcomes of AIH and AID cycles (Adjusted RR 1.128 for ongoing pregnancy rate in AIH cycles, 95% CI 0.684 to 1.860; Adjusted RR 0.751 for ongoing pregnancy rate in AID cycles, 95% CI 0.408 to 1.380). The rates of biochemical pregnancy loss (7.7 vs. 6.1%, *P* = 1.00) were similar between groups. Early miscarriage occurred 3/44 (6.8%), 4/50 (8.0%), and 1/49 (2.0%) in the exposed group of AIH cycles, the unexposed group of AIH cycles, and the unexposed group of AID cycles, respectively. Ectopic pregnancy occurred 2/44 (4.5%) in the exposed group of AIH cycles.

**Table 4:** Reproductive outcome of artificial insemination with husband or donor semen stratified by vaccine exposed or not.

**Variables**	**Exposed cycles, % (*n*)**	**Unexposed cycles, % (*n*)**	***P–*value**	Adjusted*	
				**RR (95% CI)**	***P–*value**
AIH					
Biochemical pregnancy	13.1 (44/335)	12.8 (50/390)	0.90	1.085 (0.688–1.711)	0.73
Clinical pregnancy	12.5 (42/335)	11.3 (44/390)	0.60	1.189 (0.740–1.912)	0.47
Ongoing pregnancy	11.0 (37/335)	10.3 (40/390)	0.73	1.128 (0.684–1.860)	0.64
Biochemical pregnancy loss	4.5 (2/44)	12.0 (6/50)	0.28^#^		
Early miscarriage	6.8 (3/44)	8.0 (4/50)	1.00^#^		
Ectopic pregnancy	4.5 (2/44)	0 (0)	0.22^#^		
AID					
Biochemical pregnancy	22.6 (26/115)	30.6 (49/160)	0.14	0.721 (0.401–1.295)	0.27
Clinical pregnancy	20.9 (24/115)	28.8 (46/160)	0.19	0.759 (0.416–1.383)	0.37
Ongoing pregnancy	20.9 (24/115)	28.1 (45/160)	0.17	0.751 (0.408–1.380)	0.36
Biochemical pregnancy loss	7.7 (2/26)	6.1 (3/49)	1.00^#^		
Early miscarriage	0 (0)	2.0 (1/49)	1.00^#^		
Ectopic pregnancy	0 (0)	0 (0)	1.00^#^		

^*^Adjusted for female age, BMI, infertility duration, treatment cycle type, IUI indication, sperm parameters after processing, ovarian reserve function, dominant follicles, and endometrial thickness on the day of hCG administration.

^#^Fisher exact test was used.

Subgroup analysis of vaccination status among vaccinated women in AIH cycles on reproductive outcomes was performed. As presented in Table 5, the reproductive outcomes were slightly poor in the group taken double dose or more inactivated COVID-19 vaccines than the group that took a single dose vaccine prior to intrauterine insemination, but this difference was not statistically significant (10.1 vs. 14.1% for ongoing pregnancy rate, *P* = 0.33; 11.3 vs. 16.7% for clinical pregnancy rate, *P* = 0.21; and 6.7 vs. 7.1% for miscarriage rate, *P* = 1.00). Similarly, the reproductive outcomes were slightly poor in the group that had undergone intrauterine insemination more than 3 months later after taking the last dose of COVID-19 vaccine than the group that within 3 months (9.9 vs. 14.0% for ongoing pregnancy rate, *P* = 0.29; 11.2 vs. 16.1% for clinical pregnancy rate, *P* = 0.22; and 11.1 vs. 0% for miscarriage rate, *P* = 0.27).

**Table 5:** Subgroup analysis of reproductive outcomes of artificial insemination with husband within exposed cycles.

	**Biochemical**	**Clinical**	**Ongoing**	**Biochemical**	**Miscarriage**
	**pregnancy**	**pregnancy**	**pregnancy**	**pregnancy loss**	
Doses of vaccination, % (*n*)					
Single dose prior to insemination	17.9 (14/78)	16.7 (13/78)	14.1 (11/78)	7.1 (1/14)	7.1 (1/14)
Double dose or more prior to insemination	11.7 (30/257)	11.3 (29/257)	10.1 (26/257)	3.3 (1/30)	6.7 (2/30)
*P-*value	0.15	0.21	0.33	0.54*	1.00*
Interval between the last dose and insemination					
<3 months	18.3 (17/93)	16.1 (15/93)	14.0 (13/93)	11.7 (2/17)	0 (0)
≥3 months	11.2 (27/242)	11.2 (27/242)	9.9 (24/242)	0 (0)	11.1 (3/27)
*P-*value	0.08	0.22	0.29	0.14*	0.27*

^*^Fisher exact test was used.

The predictors in the GEE model for ongoing pregnancy are presented in Table 6. After controlling bias from multiple cycles within the same couple, no independent influence factor was found to predict the reproductive outcome of AIH cycles, including COVID-19 vaccine exposed.

**Table 6:** Adjusted binary logistic regression model for predictors of ongoing pregnancy of artificial insemination with husband semen (725 cycles in 492 couples) using generalized estimating equations.

**Factor**	**Adjusted RR (95% CI)**	***P-*value**
Female vaccine exposed	1.060 (0.591–1.901)	0.85
Male partner vaccinated	0.729 (0.370–1.435)	0.36
Female age, y	1.022 (0.957–1.092)	0.51
BMI, kg/m^2^	0.968 (0.904–1.036)	0.34
Infertility duration, m	0.953 (0.824–1.103)	0.52
Treatment cycle type		
Natural	Ref.	
COS	0.684 (0.421–1.111)	0.13
IUI indication		
Unexplained/other	Ref.	
Male factors	0.870 (0.530–1.427)	0.58

## Discussion

This cohort study was designed to identify potential detrimental effects of the inactivated COVID-19 vaccine on female fertility during IUI cycles and found no significant effects on clinical pregnancy rates in either AIH or AID cycles.

The public health impact of the COVID-19 pandemic is beyond everybody's imagination 2 years after the first case was reported. China's early physical epidemic prevention measures, such as strictly blocking the transmission chain of SARS-CoV-2, have achieved great success in limiting the domestic epidemic of COVID-19. Given the integration of the world economy, and the need to open the country to the outside world, the full implementation and promotion of vaccination was the only solution. However, any resulting reproductive issues must be known and considered by reproductive medical workers. To date, there have been no reports of female reproductive system damage in COVID-19 patients, but indirect evidence suggests that COVID-19 may infect female ovarian tissue and granulosa cells through ACE2 receptors, reducing ovarian function and oocyte quality (9, 19, 20). Based on existing research on the potential impact of SARS-CoV-2 infection on female fertility, national guidelines recommend that women with pregnancy planning be actively vaccinated against COVID-19. However, these recommendations have not been accepted by the population. On the one hand, our follow-up data showed that the vaccination coverage of COVID-19 is far from establishing herd immunity in couples undergoing ART (21). On the other hand, Flynn et al. (22) investigated the impact of the COVID-19 pandemic on human pregnancy-planning behaviors through an online questionnaire and found that 53% of subjects reported that COVID-19 had affected their pregnancy plans, among which 72% chose to postpone pregnancy. These abnormal behaviors may be attributed to the lack of knowledge about the potential effects of COVID-19 vaccination, which led to much apprehension and caution among patients planning to conceive.

Current vaccines have already advanced into clinical trials, and published data mainly include inactivated virus vaccines, virus-vectored vaccines, and mRNA vaccines. The latter two were gene-based vaccines that deliver genes encoding viral antigens to host cells for *in vivo* production, which target a single protein or protein fragments of SARS-CoV-2 (23). In contrast, inactivated virus vaccines are physically or chemically inactivated but preserve the integrity of the virus particle, using the whole virus as vaccine targets. The targeted immune response of an inactivated vaccine is usually humoral and cellular, with little reactogenicity, resulting in a high safety profile (4). As for mRNA vaccines, several studies have indirectly illustrated their safety in terms of fertility. A recent report using the v-safe safety monitoring system data showed that 4,800 people had a positive pregnancy test after receiving the first dose of an mRNA COVID-19 vaccine (24). A randomized, blinded Pfizer-BioNTech trial also showed a similar number of women conceived after receiving the vaccine as those who received the placebo (15). Morris et al. (25) found no difference in implantation rates among SARS-CoV-2 vaccine seropositive, infection seropositive, and seronegative women following *in vitro* fertilization frozen embryo transfer cycles. Similarly, two observational studies have assessed the influence of the mRNA SARS-CoV-2 vaccine (BNT162b2) on IVF treatments, and neither the before-after study (26) nor the cohort study (27) demonstrated any detrimental effect on the patients' performance and ovarian reserve in IVF cycles. In addition, researchers found no significant changes in sperm characteristics before or after two doses of a COVID-19 mRNA vaccine among cohorts of healthy men (28, 29). Despite these findings, investigations into the effect of inactivated COVID-19 vaccines, the main vaccine used in China, have not been done.

This study is the first to evaluate the possible effect of inactivated COVID-19 vaccines on human reproduction, using the IUI cycle as a model. This is an effective method to study the impact of one factor on implantation, on the one hand, the fertilization process of it is relatively natural compared to IVF-ET, on the other hand, the process of IUI bypass many of the variables that normally impact the ability to conceive like ovulation and sperm selection compared to natural conception process (30). When grouping the subjects, we played close attention to the relative time between vaccination and insemination and chose a more rigorous grouping method instead of just dividing people into vaccinated and unvaccinated groups. We classify people vaccinated after insemination as vaccine unexposed group because at that time the vaccine could be considered no longer affect the process of early pregnancy. Besides, the follow-up period of our study was the period when vaccination was just started in China, at that time, sperm samples stored in the sperm bank must have come from unvaccinated donors. Since the sperm samples in AID cycles were from the sperm bank, the donor can be regarded as not affected by the vaccine. Therefore, the AID cycle is a particularly effective model for studying the effect of vaccines on female fertility by excluding any interference of male vaccination on reproductive outcomes. Although our data showed a 25% reduction in ongoing pregnancy rates in the vaccine exposure group compared to the control group during the AID cycle, there was no significant difference. The small sample size in this group limited the statistical efficacy of the AID cycle and was unable to provide conclusive results with the existing data set. However, considering the unique features of the AID cycle compared with the AIH cycle, relevant clinical data are still listed for researchers' reference.

There are several limitations of this study. Firstly, the sample size was too small to allow an in-depth stratified analysis of vaccination status with a convincing conclusion, and this will be rectified in future studies. Secondly, retrospective studies are subject to bias, and although variables linked to IUI success in prior studies were included in the GEE analysis, it was impossible to identify and control for all confounding variables. Thirdly, the participants in the present study were women undergoing ART treatments and do not represent those undergoing natural conception. Finally, due to the lack of data on the timing of male vaccination, it was impossible to judge whether the husband had been vaccinated before IUI treatment, which may lead to inaccurate results even after adjusting for the vaccination status of male partner in AIH cycles. However, these defects were partly compensated by data from AID cycles because it was known that the semen from the sperm bank had no vaccine exposure.

## Conclusions

This study provides a unique contribution to the effect of inactivated COVID-19 vaccine on female ability to conceive under a relatively rigorous design, including choosing IUI cycles as the fertility model, strict inclusion and exclusion criteria, and the application of GEE adjusted for confounding covariates based on an extensive data set of baseline and in-cycle characteristics. The present study shows no negative effects on female fertility in IUI cycles following exposure to the inactivated COVID-19 vaccine. These findings indirectly reflect the safety of inactivated COVID-19 vaccine toward reproductive health and add an extra step toward reducing vaccine hesitancy (31) among people planning to conceive.

## Data availability statement

The raw data supporting the conclusions of this article will be made available by the authors, without undue reservation.

## Ethics statement

The studies involving human participants were reviewed and approved by Ethics Committee of the Third Affiliated Hospital of Guangzhou Medical University. Written informed consent for participation was not required for this study in accordance with the National Legislation and the Institutional requirements.

## Author contributions

ZX designed the study, carried out data analysis, and drafted the manuscript. YW and YL participated in data analysis, collected all relevant data, and assisted in study conception and design. JL and HL conceived the study, participated in its design and coordination, and helped draft the manuscript. All authors contributed to the article and approved the submitted version.

## Funding

This research was supported by the National Key Research and Development Program of China (No. 2018YFC1003803 to JL), National Natural Science Foundation of China (No. 81801532 to HL), and the Guangzhou Science and Technology Plan Project (No. 202102010076 to HL). The funders had no role in the collection, analysis, and interpretation of data; in the writing of the report; and in the decision to submit the article for publication.

## Conflict of interest

The authors declare that the research was conducted in the absence of any commercial or financial relationships that could be construed as a potential conflict of interest.

## Publisher's note

All claims expressed in this article are solely those of the authors and do not necessarily represent those of their affiliated organizations, or those of the publisher, the editors and the reviewers. Any product that may be evaluated in this article, or claim that may be made by its manufacturer, is not guaranteed or endorsed by the publisher.

## Abbreviations

ART: Artificial reproductive technology
COVID-19: Coronavirus disease 2019
IUI: Intrauterine insemination
AIH: Artificial insemination with their husband's sperm
AID: Artificial insemination with donor sperm
WHO: World Health Organization
SARS-CoV-2: Severe acute respiratory syndrome coronavirus 2
ACE2: Angiotensin-converting enzyme 2
ESHRE: European Society of Human Reproduction and Embryology
ASRM: American Society for Reproductive Medicine
CDC: Centers for Disease Control and Prevention
JCVI: Joint Committee on Vaccination and Immunization
IVF-ET: *In vitro* fertilization embryo transfer
AFC: Antral follicular count
AMH: Anti-Müllerian hormone
BMI: Body mass index
COS: Controlled ovarian stimulation
FSH: Follicle-stimulating hormone
PR: progressive motility
TMSC: Total motile sperm count
HMG: Human menopausal gonadotropin
LH: Luteinizing hormone
hCG: Human chorionic gonadotropin
IQR: Interquartile range
SD: Standard deviation
RR: Risk ratio
CI: Confidence interval
GI: Generalized estimating equation.

## References

[1] Pavel S Yetiskin H Uygut MA Aslan AF Aydin G Inan Ö. Development of an Inactivated Vaccine against SARS CoV-2. Vaccines. (2021) 9:1266. 10.3390/vaccines9111266 34835197 PMC8624180

[2] World Health Organization. COVID-19 Weekly Epidemiological Update. (2022). Available online at: https://www.who.int/publications/m/item/weekly-epidemiological-update-on-covid-19-−15-march-2022 (accessed March 15, 2022).

[3] Lu R Zhao X Li J Niu P Yang B Wu H. Genomic characterization and epidemiology of 2019 novel coronavirus: implications for virus origins and receptor binding. Lancet. (2020) 395:565–74. 10.1016/S0140-6736(20)30251-8 32007145 PMC7159086

[4] Nagy A Alhatlani B. An overview of current COVID-19 vaccine platforms. Comput Struct Biotechnol J. (2021) 19:2508–17. 10.1016/j.csbj.2021.04.061 33936564 PMC8076774

[5] World Health Organization. COVID-19 Vaccine Tracker and Landscape. (2022). Available online at: https://www.who.int/publications/m/item/draft-landscape-of-covid-19-candidate-vaccines (accessed March 15, 2022).

[6] National Health Commission of the People's Republic of China. COVID-19 *Vaccination Status*. (2022). Available online at: http://www.nhc.gov.cn/xcs/yqfkdt/202112/222ad3cd759c4662a59860c2e89ca584.shtml (accessed March 15, 2022).

[7] Huang Y Yang C Xu XF Xu W Liu SW. Structural and functional properties of SARS-CoV-2 spike protein: potential antivirus drug development for COVID-19. Acta Pharmacol Sin. (2020) 41:1141–9. 10.1038/s41401-020-0485-4 32747721 PMC7396720

[8] Wang Z Xu X. scRNA-seq profiling of human testes reveals the presence of the ACE2 receptor, A target for SARS-CoV-2 infection in spermatogonia, leydig and sertoli cells. Cells. (2020) 9:920. 10.3390/cells9040920 32283711 PMC7226809

[9] Reis FM Bouissou DR Pereira VM Camargos AF dos Reis AM Santos RA. Angiotensin-(1-7), its receptor Mas, and the angiotensin-converting enzyme type 2 are expressed in the human ovary. Fertil Steril. (2011) 95:176–81. 10.1016/j.fertnstert.2010.06.060 20674894

[10] Xia S Zhang Y Wang Y Wang H Yang Y Gao GF. Safety and immunogenicity of an inactivated SARS-CoV-2 vaccine, BBIBP-CorV: a randomized, double-blind, placebo-controlled, phase 1/2 trial. Lancet Infect Dis. (2021) 21:39–51. 10.1016/S1473-3099(20)30831-8 33069281 PMC7561304

[11] Gao Q Bao L Mao H Wang L Xu K Yang M. Development of an inactivated vaccine candidate for SARS-CoV-2. Science. (2020) 369:77–81. 10.1126/science.abc1932 32376603 PMC7202686

[12] Wu Z Hu Y Xu M Chen Z Yang W Jiang Z. Safety, tolerability, and immunogenicity of an inactivated SARS-CoV-2 vaccine (CoronaVac) in healthy adults aged 60 years and older: a randomized, double-blind, placebo-controlled, phase 1/2 clinical trial. Lancet Infect Dis. (2021) 21:803–12. 10.1016/S1473-3099(20)30987-7 33548194 PMC7906628

[13] Xia S Duan K Zhang Y Zhao D Zhang H Xie Z. Effect of an inactivated vaccine against SARS-CoV-2 on safety and immunogenicity outcomes: interim analysis of 2 randomized clinical trials. JAMA. (2020) 324:951–60. 10.1001/jama.2020.15543 32789505 PMC7426884

[14] European Society of Human Reproduction Embryology. COVID-19 Vaccination and Assisted Reproduction. (2021). Available online at: https://www.eshre.eu/Home/COVID19WG. Embryology ESoHRa. COVID-19 vaccination and assisted reproduction (accessed March 15, 2022).

[15] American Society for Reproductive Medicine. UPDATE No. 18-COVID-19 Vaccination, Booster Shots Reproductive Health Care. (2021). Available online at: https://www.asrm.org/news-and-publications/news-and-research/press-releases-and-bulletins/update-no-18-covid-19-vaccination-booster-shots-and-reproductive-health-care/ (accessed March 15, 2022).

[16] Ayeleke RO Asseler JD Cohlen BJ Veltman-Verhulst SM. Intra-uterine insemination for unexplained subfertility. Cochrane Database Syst Rev. (2020) 3:CD001838. 10.1002/14651858.CD001838.pub6 32124980 PMC7059962

[17] Zarek SM Hill MJ Richter KS Wu M DeCherney AH Osheroff JE. Single-donor and double-donor sperm intrauterine insemination cycles: does double intrauterine insemination increase clinical pregnancy rates. Fertil Steril. (2014) 102:739–43. 10.1016/j.fertnstert.2014.05.018 24934490 PMC4149942

[18] Li S He Y Cao M Liu H Liu J. Low-dose human menopausal gonadotrophin versus natural cycles in intrauterine insemination for subfertile couples with regular menstruation. J Ovarian Res. (2020) 13:36. 10.1186/s13048-020-00638-3 32247312 PMC7129328

[19] Vaz-Silva J Carneiro MM Ferreira MC Pinheiro SV Silva DA Silva-Filho AL. The vasoactive peptide angiotensin-(1-7), its receptor Mas and the angiotensin-converting enzyme type 2 are expressed in the human endometrium. Reprod Sci. (2009) 16:247–56. 10.1177/1933719108327593 19164480

[20] Barreta MH Gasperin BG Ferreira R Rovani M Pereira GR Bohrer RC. The components of the angiotensin-(1-7) system are differentially expressed during follicular wave in cattle. J Renin Angiotensin Aldosterone Syst. (2015) 16:275–83. 10.1177/1470320313491996 23764714

[21] Anderson RM Vegvari C Truscott J Collyer BS. Challenges in creating herd immunity to SARS-CoV-2 infection by mass vaccination. Lancet. (2020) 396:1614–6. 10.1016/S0140-6736(20)32318-7 33159850 PMC7836302

[22] Flynn AC Kavanagh K Smith AD Poston L White SL. The impact of the COVID-19 pandemic on pregnancy planning behaviors. Womens Health Rep. (2021) 2:71–7. 10.1089/whr.2021.0005 33786533 PMC8006747

[23] Dai L Gao GF. Viral targets for vaccines against COVID-19. Nat Rev Immunol. (2021) 21:73–82. 10.1038/s41577-020-00480-0 33340022 PMC7747004

[24] Shimabukuro TT Kim SY Myers TR Moro PL Oduyebo T Panagiotakopoulos L. preliminary findings of mRNA COVID-19 vaccine safety in pregnant persons. N Engl J Med. (2021) 384:2273–82. 10.1056/NEJMoa2104983 33882218 PMC8117969

[25] Morris RS. SARS-CoV-2 spike protein seropositivity from vaccination or infection does not cause sterility. F S Rep. (2021) 2:253–5. 10.1016/j.xfre.2021.05.010 34095871 PMC8169568

[26] Orvieto R Noach-Hirsh M Segev-Zahav A Haas J Nahum R Aizer A. Does mRNA SARS-CoV-2 vaccine influence patients' performance during IVF-ET cycle. Reprod Biol Endocrinol. (2021) 19:69. 10.1186/s12958-021-00757-6 33985514 PMC8116639

[27] Bentov Y Beharier O Moav-Zafrir A Kabessa M Godin M Greenfield CS. Ovarian follicular function is not altered by SARS-CoV-2 infection or BNT162b2 mRNA COVID-19 vaccination. Hum Reprod. (2021) 36:2506–13. 10.1093/humrep/deab182 34364311 PMC8385874

[28] Gonzalez DC Nassau DE Khodamoradi K Ibrahim E Blachman-Braun R Ory J. sperm parameters before and after COVID-19 mRNA vaccination. JAMA. (2021) 326:273–4. 10.1001/jama.2021.9976 34137808 PMC8293015

[29] Lifshitz D Haas J Lebovitz O Raviv G Orvieto R Aizer A. Does mRNA SARS-CoV-2 vaccine detrimentally affect male fertility, as reflected by semen analysis. Reprod Biomed Online. (2021). 10.1016/j.rbmo.2021.09.021 PMC8489287 34815157

[30] Leung E Lee CL Tian X Lam K Li R Ng E. Simulating nature in sperm selection for assisted reproduction. Nat Rev Urol. (2021). 10.1038/s41585-021-00530-9 34741158

[31] Merkley E Loewen PJ. Assessment of communication strategies for mitigating COVID-19 vaccine-specific hesitancy in Canada. JAMA Netw Open. (2021) 4:e2126635. 10.1001/jamanetworkopen.2021.26635 34591105 PMC8485173

